# The Use of Succinonitrile as an Electrolyte Additive for Composite-Fiber Membranes in Lithium-Ion Batteries

**DOI:** 10.3390/membranes10030045

**Published:** 2020-03-17

**Authors:** Jahaziel Villarreal, Roberto Orrostieta Chavez, Sujay A. Chopade, Timothy P. Lodge, Mataz Alcoutlabi

**Affiliations:** 1 Department of Mechanical Engineering, University of Texas, Rio Grande Valley, Edinburg, TX 78539, USA; jahaziel.villarreal@lynntech.com (J.V.); roberto.orrostietachavez01@utrgv.edu (R.O.C.); 2 Department of Chemical Engineering and Materials Science and Department of Chemistry, University of Minnesota, Minneapolis, MN 55455, USA; chopade123@gmail.com (S.A.C.); lodge@umn.edu (T.P.L.)

**Keywords:** ionic liquids, succinonitrile, electrolyte, lithium ion batteries, composite fibers, mixtures

## Abstract

In the present work, the effect of temperature and additives on the ionic conductivity of mixed organic/ionic liquid electrolytes (MOILEs) was investigated by conducting galvanostatic charge/discharge and ionic conductivity experiments. The mixed electrolyte is based on the ionic liquid (IL) (EMI/TFSI/LiTFSI) and organic solvents EC/DMC (1:1 *v*/*v*). The effect of electrolyte type on the electrochemical performance of a LiCoO_2_ cathode and a SnO_2_/C composite anode in lithium anode (or cathode) half-cells was also investigated. The results demonstrated that the addition of 5 wt.% succinonitrile (SN) resulted in enhanced ionic conductivity of a 60% EMI-TFSI 40% EC/DMC MOILE from ~14 mS·cm^−1^ to ~26 mS·cm^−1^ at room temperature. Additionally, at a temperature of 100 °C, an increase in ionic conductivity from ~38 to ~69 mS·cm^−1^ was observed for the MOILE with 5 wt% SN. The improvement in the ionic conductivity is attributed to the high polarity of SN and its ability to dissolve various types of salts such as LiTFSI. The galvanostatic charge/discharge results showed that the LiCoO_2_ cathode with the MOILE (without SN) exhibited a 39% specific capacity loss at the 50th cycle while the LiCoO_2_ cathode in the MOILE with 5 wt.% SN showed a decrease in specific capacity of only 14%. The addition of 5 wt.% SN to the MOILE with a SnO_2_/C composite-fiber anode resulted in improved cycling performance and rate capability of the SnO_2_/C composite-membrane anode in lithium anode half-cells. Based on the results reported in this work, a new avenue and promising outcome for the future use of MOILEs with SN in lithium-ion batteries (LIBs) can be opened.

## 1. Introduction

Lithium-ion batteries (LIBs) are widely used in electronic devices ever since their successful commercialization by Sony in 1991 and Asahi Kasei and Toshiba in 1992 [1,2]. The conventional electrolyte used in LIBs is based on lithium hexafluorophosphate (LiPF_6_) salt dissolved in volatile organic solvents; typically, these are mixtures of carbonates such as ethylene carbonate (EC), ethyl methyl carbonate (EMC), diethyl carbonate (DEC), and dimethyl carbonate (DMC) [3,4]. These combinations enhance desired properties in electrolytes. For example, EC has a high dielectric constant that promotes salt dissolution, and the addition of DMC to these organic solvents can lower the melting point and viscosity of the combined EC/DMC organic liquid electrolyte (OLE) [5]. Despite the high ionic conductivity and Li-ion diffusivity of OLEs during the charge/discharge cycles in LIBs, they face serious safety concerns due to their high flammability and volatility [6]. Such safety hazards can lead to thermal runaway and serious consequences [7,8]. Recently, incidents involving violent battery ignitions have caught the general public’s attention, and this concern has increased even more with the application of lithium-ion batteries in electric vehicles and power grid storage devices [9]. Because ionic liquid electrolytes (ILEs) are non-flammable, non-volatile, and conductive, they possess safety advantages over OLEs and have been studied as electrolytes in rechargeable LIBs [10,11]. ILEs tend to be electrochemically and thermally stable, potentially allowing use of high voltage cathodes and safe operation at high temperatures. Nonetheless, ILEs face many challenges including larger viscosity, which increases significantly with decreasing temperature, crystallization at low temperatures, and large, strongly temperature-dependent interfacial impedance at both the cathode and anode. All of these issues are tied to the fact that the ILE-based Li-ion cells that have been developed to date operate only at elevated temperatures and at relatively low charging/discharging rates [10]. The high viscosity of ILEs typically results in a decreased total ionic conductivity at room temperature. Most important for electrolyte performance is to maximize the conductivity carried by the Li+ (cation), i.e., the product of total conductivity and transference number [12]. Additionally, it has been observed in both experiments and simulations that Li+ mobility increases more rapidly with dilution with an organic solvent than do the mobilities of the RTIL anion and especially cation, resulting in a higher transference number [13,14]. We note the further complication that the ability of a low viscosity solvent to dissolve lithium salt and transport Li^+^ does not necessarily imply improved electrolyte performance [15,16].

A novel approach based on mixing organic solvents with ILEs has been used to improve the ionic conductivity of ILEs in Li-ion cells with the aim to offer combined advantages of OLs and ILEs such as decreased viscosity, higher Li+ diffusion/mobility, (i.e., improved conductivity), and, under appropriate volume ratios, better safety factors [6]. An increased tolerance to higher operational temperatures is beneficial not only because batteries are less likely to experience thermal runaway, but also because it enables specialty applications that require very low (−80 °C to −40 °C) or high (100 °C) temperatures; electrolyte crystallization can be prevented at lower working temperature conditions by modifying the IL solvent, while battery performance at higher temperature can be achieved by adding different lithium salts to ILs [17]. Several IL/Li salt systems have been employed as electrolytes in Li-ion batteries, but the number of ILEs that have been demonstrated as effective in operating cells is limited. Since demonstrating reasonable conductivity of an ILE (commonly done) and reasonable electrochemical stability (less commonly undertaken) are not sufficient to ensure reliable operation of a battery, a number of other issues such as interfacial defects must be considered. As a result, the number of operating batteries based on ILEs is less extensive than might be expected, given the huge number of anode/cathode and electrolyte combinations possible [11,18,19,20]. 

The most common anion investigated for ILEs is bis(trifluoromethanesulfonyl)-imide (TFSI), while the most common cations are alkylimidazoliums, tetraalkylammoniums, and alkylpryrrolidiniums (e.g., pyr13 and pyr14) [11]. 1-Ethyl-3-methylimidazolium bis(trifluoromethanesulfonyl)-imide (EMI-TFSI) has been used with Li salt as an electrolyte for LIBs due to its low viscosity compared to other ILEs [21]. For example, a LiCoO_2_ cathode in a (EMI/TFSI + LiTFSI) electrolyte delivered higher discharge capacity at room temperature than in the EMIBF_4_ + LiBF_4_ system [21]. EMI-TSFI is employed here as an IL due to its good conductivity (8.7–9.1 mS·cm^−1^ at 25 °C), low viscosity (33–34 cP), and low melting point (−15 °C) [17]. Additionally, it has been found that EMI-TSFI with LiTSFI can increase the ionic conductivity and decrease the viscosity of the electrolyte, while no flammability was observed for compositions with IL (EMI-TSFI) wt% of 40% or more in EC–DEC–VC–1M LiPF_6_ electrolytes at increased temperatures [21,22]. 

Reversible capacities of up to 155 mAh g^−1^ and 128 mAh g^−1^ have been reported for Li-ion full cells with EMI-TSFI and EC/DMC MOILEs using LiFePO_4_ as the cathode with a graphite anode, and a LiFePO_4_ cathode with a Li_4_Ti_5_O_12_ anode, respectively [22]. Nonetheless, the addition of non-ionic organic additives such as succinonitrile (SN) to the electrolyte can improve the ionic conductivity of ILEs and the overall electrochemical performance of the Li-ion cell [1,23]. SN can dissolve different types of salts such as LiTFSI, LiBF_4_, LiPF_6_, LiN(CN)_2_, Ba(TFSI)_2_, Pb(TSFI)_2_, La(TSFI)_2_, Ag(CF_3_SO_3_), and Cu(CF_3_SO_3_) [24]. SN has been frequently used as a solid electrolyte in LIBs [24,25,26,27,28] but limited results have been reported on the use of SN with OLEs and ILEs in LIBs [29]. For example, the addition of SN to a polymer electrolyte (PEO-LiTFSI, P(VDFHFP)– LiTFSI, and P(VDF-HFP)–LiBETI) resulted in improved ionic conductivity of the polymer electrolyte and favorable mechanical properties [30]. SN has been recently used as a functional additive to improve the thermal stability and broaden the oxidation electrochemical window of an OLE in lithium cathode half-cells containing a LNMO cathode. The results showed that the addition of SN to the electrolyte solution lead to a remarkably improved cycling stability, which was due to the formation of an electronically conductive film on the cathode [29,30]. SN was also used as an additive to improve the thermal stability of ethylene carbonate (EC)-based electrolytes in LIBs. This work showed that SN can suppress parasitic reactions between the positive electrode (LiCoO_2_) and the organic liquid electrolyte, because the nitrogen ion in the nitrile functional group (–CN) in SN has a lone pair of electrons leading to a strong bond with the transition metal ions on the cathode. It was also reported that the addition of SN resulted in a suppression of electrolyte decomposition in commercial cells [30]. The authors also suggested that SN can react with transition metal ions in the electrolyte to form metal ion compounds preventing their reduction on the negative electrode surface, which would compromise the SEI surface. This work focuses on the investigation of the electrochemical properties of EMI-TFSI-LiTFSI electrolytes in lithium anode (or cathode) half-cells using either a cathode or an anode [31]. OLE (EC/DMC 1:1 *v*/*v*), ILE (EMI-TFSI), and MOILE were used with a commercial cathode, LiCoO_2_, and a SnO_2_/C composite-fiber anode in lithium anode half-cells to investigate the effect of electrolyte type on the electrochemical performance. The effects of temperature and SN additive on the ionic conductivity and electrochemical performance of MOILEs were investigated by conducting charge/discharge and impedance measurements on the lithium anode (or cathode) half-cells with commercial cathode materials. 

## 2. Experimental

### 2.1. Materials

Bis(trifluoromethane)sulfonimide lithium salt (Li-TFSI) (99%) and 1-ethyl-3-methylimidazolium bromide (EMI-Br) (99.98%) were purchased from Io-li-tech, Tuscaloosa, AL, USA. Ethylene carbonate (EC) (99%) and dimethyl carbonate (DMC) were purchased from Alpha Aesar (Tewksbury, MA, USA) and Fisher Scientific (Lenexa, KS, USA), respectively. LiTFSI (98%), HPLC water, silicon oxide, dichloromethane (DCM) (99%), and succinonitrile (SN, 99%) were obtained from Sigma-Aldrich (St. Louis, MO, USA)). Polyacrylonitrile (PAN) with *M*_w_ ≈ 150,000 was purchased from Sigma-Aldrich. Dimethylformamide (DMF) (>99.5%), and tin (II) 2-ethylhexaonate were purchased from Fisher Scientific.

### 2.2. Electrolyte Preparation

1-Ethyl-3-methyl-imidazolium bis(trifluoromethanesulfonyl)imide (EMI-TFSI), was synthesized by reacting HPLC water with a 1:1 LiTFSI:EMI-Br molar ratio mixture. This solution was stirred for 24 h in an oil bath at 70 °C. After the reaction took place, an aqueous layer and an ionic liquid (EMI-TFSI) rich layer were formed, and the solution was extracted from the oil bath. Once the solution was cooled to room temperature, the EMI-TFSI was separated from its aqueous counterpart and decanted into a separator funnel. HPLC water was poured into the separator funnel and mixed with the EMI-TFSI. The mixture was left to rest until the two layers were formed again. The EMI-TFSI layer was once again removed. This process was repeated two more times. Then, the EMI-TFSI was placed in a 500 mL round bottom flask to be dissolved with a sufficient amount of DCM. The dissolved EMI-TSFI was decanted into a chromatography column in order to filter any remaining impurities. The chromatography column contained one inch of sand, followed by silica oxide fully covering the remaining of the column up to the beginning of reservoir. The collected solution was then placed in a rotavap to remove the solvent (DCM) from the EMI-TSFI. Finally, the obtained EMI-TSFI was placed in a vacuum oven at 100 °C for 48 h to remove any water and excess DCM. The purity of the synthesized IL electrolyte was confirmed by ^1^H NMR spectroscopy.

The organic liquid electrolyte was prepared in a glove box (MBRAUN, Garching, Germany) with a controlled argon atmosphere. A 20 mL solution was prepared by combining a 1:1 *v*/*v* ratio of EC and DMC followed by 2 h of magnetic stirring. This OLE solution was stored and used to make 5 mL batches of 1 M LiTFSI in 60% EMI-TFSI and 40% EC/DMC. First, 1.435 g of LiTFSI and 2.564 g of EC/DMC solution were stirred until the LiTFSI was fully dissolved. Next, 4.590 g of EMI-TFSI were added and stirred for 24 h. The final weight of the solution was 8.590 g. Using this weight, an additional 0.429 g of SN was added to compare the ionic conductivity of the MOILE with one containing 5 wt.% of SN. 

The ionic conductivity of the MOILEs was measured by assembling coin-type cells (CR2032) composed of two stainless-steel spacers as the positive and negative terminals, and a Teflon washer filled with MOILE. The LiCoO_2_ cathode was assembled with a common half-cell configuration to investigate the electrochemical performance of the cell. The as-prepared MOILE was used with the commercial LiCoO_2_ cathode. The active material loading in the electrode was 6.2–8.0 mg/cm^2^. The coin cells were assembled in a glove box using the cathode as the working electrode, with a Li counter electrode and microfiber glass mat separator (Whatman). 

### 2.3. Preparation of SnO_2_/C Composite Fiber Membranes

The SnO_2_/C composite fibers were prepared by forcespinning of PAN/SnO_2_ precursor fibers followed by a thermal treatment. The PAN/SnO_2_ solution was prepared by dissolving 12 wt% PAN in DMF. A tin (II) 2-ethylhexanoate solution to 2:1 weight ratio of PAN solution was added and stirred for 24 h. The forcespinning process relies on applying centrifugal forces at high rotational speeds to a polymer solution or melt to produce microfibers with different structure and morphology. A description of the forcespinning process was given previously [19,32,33]. The PAN/SnO_2_ precursor solution was spun using the FiberRio L-1000 cyclone at a rotational speed of 8000 rpm for 1 min. The PAN/SnO_2_ fibrous mat was collected, stabilized in air at 280 °C for 5 h, and subsequently carbonized under argon atmosphere at 700 °C for 3 h (heating rate: 3 °C/min). The SnO_2_/C composite fibers were removed from the tube furnace, punched into 0.5 in (0.0127 m) diameter anodes, then weighed and used directly as working electrodes in lithium anode half-cells. 

### 2.4. Fiber Membrane Characterization

The morphology and structure of composite fiber membranes were investigated using a scanning electron microscope (SEM) from Sigma VP Carl Zeiss, Oberkochen, Germany while energy-dispersive X-ray spectroscopy (EDS) from EDAX Inc., Mahwah, NJ, USA was used to confirm the elemental composition of the fibers. The crystal structure of the composite fiber membranes was evaluated by X-ray diffraction (XRD) using a Bruker D8 Advanced X-ray Diffractometer at a scan rate of 1 °C/min over a range of 2θ angle from 10° to 70°.

### 2.5. Electrochemical Measurements

Lithium anode (or cathode) half-cells were assembled in an argon-filled glove box with SnO_2_/C composite fibers as a binder-free anode and Li-metal as the counter electrode, using the MOILE. The electrothermal performance of the SnO_2_/C composite-fiber anode was evaluated by conducting galvanostatic charge/discharge experiments on CR2032 coin cells at 100 mA g^−1^. The active material loading in the anode was 2.4–4.5 mg/cm^2^. The ionic conductivity experiments on half cells with MOILEs were performed at different temperatures using a home-built heating block chamber. The design was based on a home-built sealed conducting cell in use at the University of Minnesota [23]. The impedance of the MOILEs at different temperatures was measured using a Metrohm Autolab (PGSTAT128N) connected to the heating chamber, over a frequency range from 0.1 Hz to 1 kHz. The ionic conductivity of the electrolyte was determined using coin cells with two stainless steel blocking electrodes filled with the electrolyte. For accurate measurements of the ionic conductivity, a Teflon spacer was placed between the stainless-steel electrodes to hold the electrolyte inside the cell. The sample (electrolyte) preparation was conducted in an argon-filled glove box. The cell was then taken outside the glove box and inserted in the heating chamber. The ionic conductivity, σ, was calculated as L/(RA), where L and A are the sample thickness and superficial area of the sample and R is the bulk resistance [23]. The bulk resistance was determined from the frequency-independent plateau of the real part of the impedance (Z′). The temperature was controlled and monitored using thermocouples and heating cartridges connected to a temperature process control CN 7500 purchased from Omega. The experimental setup was connected to a personnel computer using a RS485 USB converter to monitor the time and temperature during the impedance measurements. 

The electrochemical performance of the LiCoO_2_ half-cells was evaluated at 60 °C. The LiCoO_2_ half cells were placed in a controlled temperature oven (ESPEC BTZ – 133). LiCoO_2_ cathode with electrolytes 1 M LiTFSI in 60% EMI-TFSI and 40% EC/DMC with and without SN were tested at a current density of 100 mAh g^−1^ for 50 cycles. Arbin’s MTIS Pro was employed to conduct the galvanostatic charge/discharge experiments over a voltage range of 2.5–4.2 V. A port extension was connected between the Arbin instrument and the ESPEC oven to conduct the electrochemical experiments at different temperatures. 

## 3. Results and Discussion

### 3.1. Materials Characterization

**Figure 1:**
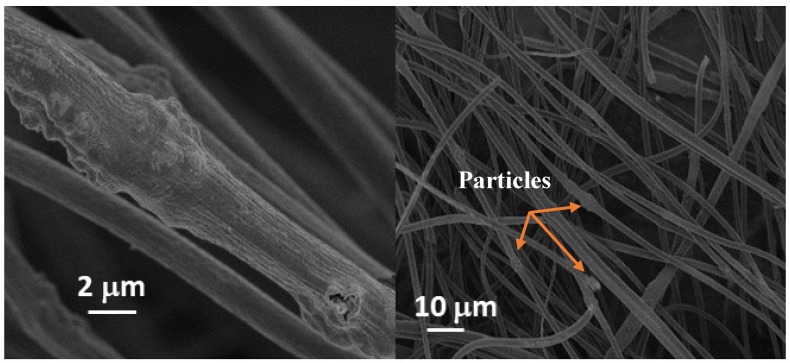
SEM images of a SnO_2_/C composite-fiber membrane [20], with copyright permission from the IOP Publishing.

Figure 1 shows SEM images of SnO_2_/C composite fibers. It can be seen in Figure 1 that the SnO_2_ nanoparticles tend to aggregate, forming large clusters on the fibers. Some of these nanoparticles are embedded in the fibers and some are deposited on the fiber strands [20]. The average fiber diameter of the SnO_2_/C composite fibers was 1.86 m.

**Figure 2:**
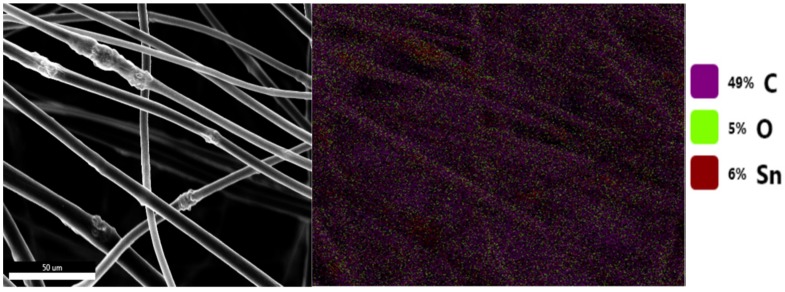
SEM image of SnO_2_/C composite fibers (left) and corresponding EDS mapping of the SnO_2_/C composite fibers (right).

Figure 2 shows an SEM image of SnO_2_/C composite fibers and the corresponding EDS mapping. Figure 2 shows that the composite fibers consist of C, O, and Sn that are distributed over the fibers. The EDS results confirm that the aggregated nanoparticles on the fibers contain Sn and O (i.e., SnO_2_ nanoparticles), which are attached to the surface of the carbon-fiber matrix 

**Figure 3:**
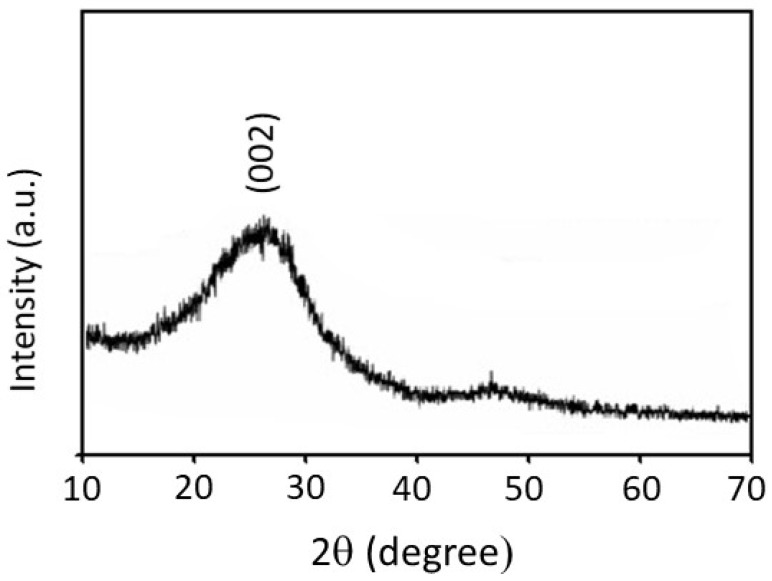
XRD pattern of the carbon-fiber membrane prepared after the carbonization of polyacrylonitrile (PAN) fibers at 700 °C.

Figure 3 shows an XRD pattern for the carbon fibers, where a broad diffraction peak is observed at 2θ = 27.8° corresponding to the (002) lattice plane of graphite [34,35,36]. It is observed in Figure 3 that this peak is weak and broad, which is the result of the formation of an amorphous carbon fiber structure after carbonization of the precursor PAN fibrous membrane.

**Figure 4:**
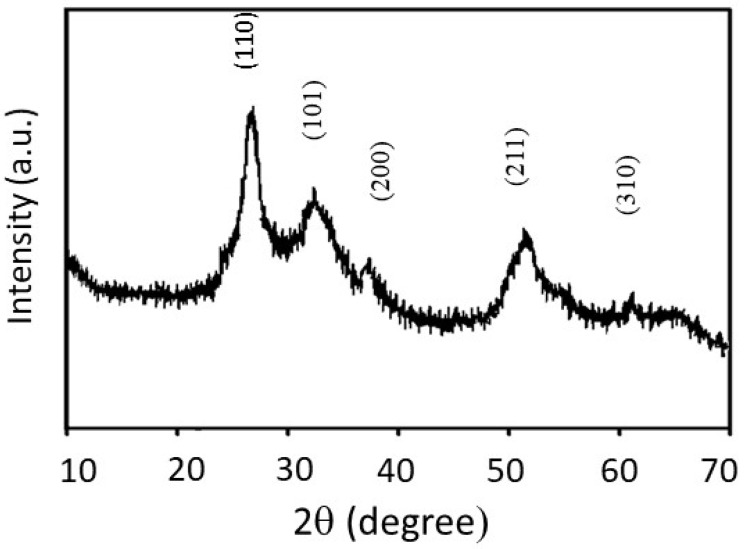
XRD pattern of the SnO_2_/C composite-fiber membrane prepared after calcination of PAN/SnO_2_ precursor fibers at 700 °C.

Figure 4 shows XRD analysis of the SnO_2_/C composite-fiber membrane. The observed pattern has predominantly crystalline peaks corresponding to (110), (101), (200), (211), and (310) planes. The observed peaks overlap with five of the seven peaks of the SnO_2_ crystal structure published by the (JCPDS 41-1445), further confirming the formation of SnO_2_ nanoparticles in the carbon matrix. 

### 3.2. Ionic Conductivity Measurement of Electrolytes at Different Temperature

**Figure 5:**
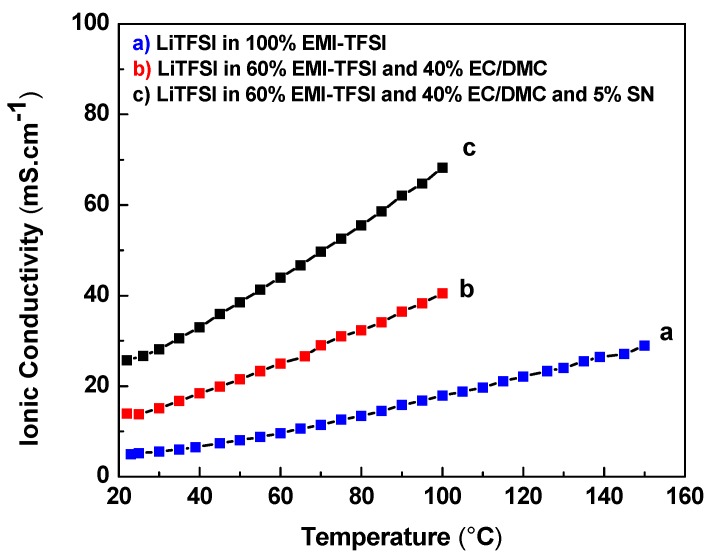
Ionic conductivity vs temperature for the ionic liquid electrolyte (ILE), mixed organic/ionic liquid electrolyte (MOILE), and MOILE with 5 wt.% SN.

Figure 5 shows the ionic conductivity of the ILE and MOILEs as a function of temperature. The ILE was prepared from LiTFSI salt dissolved in 100% EMI-TFSI while the MOILE was prepared by dissolving LiTFSI salt in 60% EMI-TFSI and 40% EC/DMC, with and without the addition of 5% SN. The results show that the ILE (100% EMI-TFSI) delivered an ionic conductivity ~5 mS·cm^−1^ at room temperature, which is lower than that for the MOILE (60% EMI-TFSI and 40% EC/DMC) (~14 mS·cm^−1^). It is also clear in Figure 5 that the ionic conductivity of the three electrolytes increases with increasing temperature. The MOILE with 5% SN shows the highest ionic conductivity at 100 °C (70 mS·cm^−1^) among these three electrolytes. Despite its lower conductivity at room temperature, the ILE ionic conductivity increased significantly as the temperature was increased. At 150 °C, the ILE conductivity was ~30 mS·cm^−1^. This behavior is expected since the viscosity of ILEs tends to decrease with increasing temperature. The addition of 40% organic liquid, EC/DMC (1:1 *v*/*v* ratio), to 1 M LiTFSI in 60% EMI-TFSI resulted in an increased ionic conductivity of ~14 mS·cm^−1^ at room temperature while the MOILE with the addition of 5 wt% SN exhibited the highest room temperature ionic conductivity of ~26 mS·cm^−1^. Note here that the ionic conductivity of the OLE (EC/DMC/LiTFSI) is not shown in Figure 5 since there are data available in the literature on LiTFSI in binary EC/DMC mixtures. In fact, LiTFSI salt in EC/DMC binary system shows a higher ionic conductivity than that for ILE and MOILE. For example, results reported by Dahbi et al. showed that the LiTFSI in EC/DMC (1:1 *v*/*v* ratio), which is the same OLE used in the present work, exhibited an ionic conductivity of 8.6 mS·cm^−^^1^ at 25 °C. This value was increased to 11.5 and 14.9 mS·cm^−^^1^ when the temperature was increased to 40 and 60 °C, respectively [37]. The results also showed the ionic conductivity of LiPF_6_ in EC/DMC mixture was higher than that with EC/DMC/LiTFSI electrolyte over the entire temperature range [37]. 

### 3.3. Electrochemical Performance of A LiCoO_2_ Electrode in Lithium Cathode Half-Cells

The commercial LiCoO_2_ electrode was employed in lithium cathode half-cells with a single-coated lithium foil to investigate its electrochemical performance. The MOILE with and without 5 wt% SN was used with the commercial LiCoO_2_. The purpose was to evaluate the behavior of the MOILEs in high voltage cathode materials such as LiCoO_2_, which has a larger voltage range than LiFePO_4_. LiCoO_2_ still dominates the portable electronics market due to its high voltage plateau and easy synthesis compared to LiFePO_4_ [38]. Galvanostatic charge/discharge experiments were performed for 50 cycles at different temperatures and at a current density of 100 mA g^−1^.

**Figure 6:**
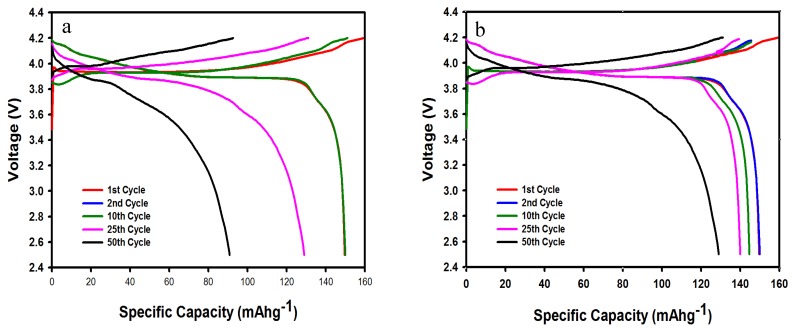
Charge/discharge profiles of a commercial LiCoO_2_ cathode at 60 °C with MOILEs (**a**) 1 M LiTFSI 60% EMI-TFSI 40% EC/DMC EC/DMC (1:1 *v*/*v*), and (**b**) 1 M LiTFSI 60% EMI-TFSI 40% EC/DMC EC/DMC (1:1 *v*/*v*) containing 5 wt% SN. Current density = 100 mA g^−1^.

Figure 6a,b shows the charge/discharge profiles at 60 °C and at 100 mA g^−1^ of the commercial LiCoO_2_ cathode in MOILEs without SN and with 5 wt% SN, respectively. As can be observed in Figure 6a, the LiCoO_2_ cathode in the MOILE without SN maintained a consistent specific capacity of 148 mAh g^−1^ up to the 10th cycle. However, significant irreversibilities were observed at the 25th and 50th cycles. After 50 cycles, the cathode delivered a discharge capacity of 91 mAh g^−1^, indicating a capacity retention of 61.5% at a current density of 100 mA g^−1^. The discharge capacity retention is equal to the capacity after the 50th cycle divided by the capacity at the 1^st^ cycle (i.e., 61.5% = (100–38.5)%). On the other hand, the LiCoO_2_ cathode in the MOILE with SN (Figure 6b) exhibited an initial discharge capacity of 150 mAh g^−1^ at 100 mA g^−1^, and after the 50th cycle, the discharge capacity reached a value of 129 mA g^−1^ indicative of acceptable capacity retention of 86%. The improvement in the electrochemical performance of the LiCoO_2_ cathode is attributed to the effect of the SN additive on the ILE, and to the high conductivity of MOILEs at high temperature (60 °C). The high volatility and evaporation (high vapor pressure) of DMC at high temperature might influence the ionic conductivity of electrolytes containing a high percentage of DMC, thus affecting the electrochemical performance of the electrode. The effect of DMC on the ionic conductivity of MOILEs was not investigated since the amount of DMC in MOILEs is only 20% (1 M LITFSI in 1:1 *v*/*v* EC/DMC) and this should affect the performance of the electrode only slightly. However, results reported in the literature show that the ionic conductivity of 1 M LiPF_6_ in DMC remains significant (i.e., 9 mS·cm^−1^) at 55 °C [39]. Results reported by Aurbach et al. on a LNMO cathode at 60 °C in a liquid electrolyte (DMC–EC (2:1)/LiPF_6_ 1.5 M), over a 3.5–4.9 V potential range showed that the cycling behavior of the cathode was explored without any observed degradation of the electrolyte solution [40]. 

It is worth noting here that the LiCoO_2_ cathode in the MOILEs shows moderate capacity fading and voltage change in the plateau of Figure 6a,b. This might be caused by a decrease in active material (lithium) on the current collector after the 25th cycle. Another important factor that could affect this loss in capacity of the LiCoO_2_ cathode is that the corrosion of the Al current collector on the cathode side by the TFSI, thereby contributing to the loss of active material from the Al current collector [41]. More work will be conducted to investigate these effects on the LiCoO_2_ cathode in LiTFSi/MOILEs systems. 

**Figure 7:**
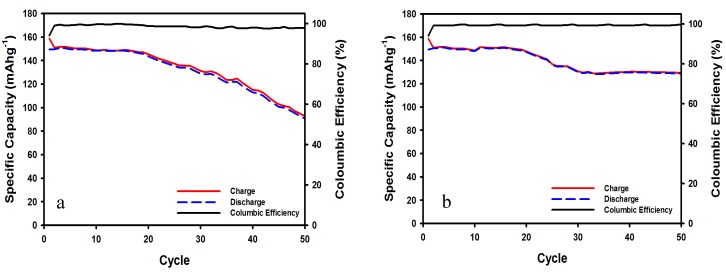
Cycling performance and coulombic efficiency of LiCoO_2_ commercial cathode at 60 °C in (**a**) 1 M LiTFSI in 60% EMI-TFSI 40% EC/DMC (1:1 *v*/*v*) electrolyte, and (**b**) 1 M LiTFSI in 60% EMI-TFSI 40% EC/DMC (1:1 *v*/*v*) with 5 wt% SN. Current density = 100 mA g^−1^.

Figure 7a,b shows the cycling performance corresponding to the charge/discharge curves shown in Figure 6a,b. Although the capacity is stable within the first twenty cycles, the LiCoO_2_ cathode in the MOILE without SN suffered from a steady loss in specific capacity after 20 cycles. In contrast, the same cathode in the MOILE with 5 wt% SN maintained a stable specific capacity for the first 20 cycles; there was a slight decrease in capacity between 20th and 30th cycles, while thereafter the cathode maintained a constant capacity of ~129 mAh g^–1^. The LiCoO_2_ cathode in both electrolytes maintained a similar high coulombic efficiency of 98% for 50 cycles. 

**Figure 8:**
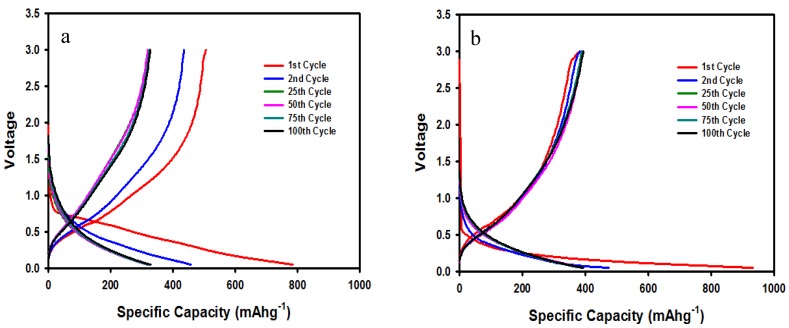
Charge/discharge profiles for a SnO_2_/C composite-fiber anode at 25 °C in two different electrolytes: (**a**) 1M LiPF_6_ in EC/DMC (1:1 *v*/*v*) electrolyte, and (**b**) 1 M LiTFSI in 60% EMI-TFSI/ 40% EC/DMC (1:1 *v*/*v*) with 5 wt% SN.

Figure 8a,b shows the charge/discharge curves at 100 mA g^−1^ for the SnO_2_/C composite-fiber anode in two different electrolytes, OLE and MOILE with SN. The cycle performance of the SnO_2_/C composite electrode was evaluated by conducing galvanostatic charge/discharge experiments at room temperature and at a current density of 100 mA g^−1^. The voltage range for lithium anode half-cells tested with the SnO_2_/C composite-fiber anode was 0.05–3 V (versus Li+/Li). The SnO_2_/C composite-fiber anode with the 1 M LiPF_6_ in EC/DMC (1:1 *v*/*v*) electrolyte showed an initial discharge capacity of 785 mAh g^−1^. The reversible specific capacity after 100 cycles was 319 mAh g^–1^. Nevertheless, the SnO_2_/C composite anode showed a stable specific capacity after the 25th cycle, with a capacity retention of 98% after the 2nd cycle. Improved cycling stability of the SnO_2_/C composite fibers in 1 M LiTFSI in 60% EMI-TFSI 40% EC/DMC with 5% SN electrolyte (Figure 8b) was observed after the 2nd cycle, with a specific capacity of 382 mAh g^−1^_,_ having a ~20% increase compared to the SnO_2_/C composite-fiber anode cycled with the OLE. 

**Figure 9:**
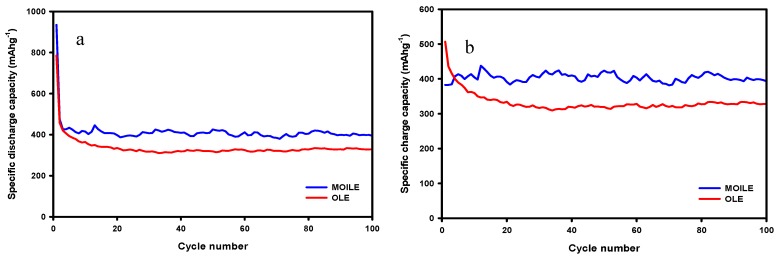
Cycling performance of a SnO_2_/C composite-fiber anode at 25 °C in two different electrolytes: (**a**) 1M LiPF_6_ in EC/DMC (1:1 *v*/*v*) electrolyte, and (**b**) 1 M LiTFSI in 60% EMI-TFSI/ 40% EC/DMC (1:1 *v*/*v*) with 5 wt% SN.

Figure 9 shows the cycling performance (charge/discharge capacity vs. cycle number) of the SnO_2_/C composite-membrane anode in the OLE and the MOILE with 5 wt% SN at a current density of 100 mA g^−1^. It is observed in Figure 9a,b that the discharge and charge capacities of the SnO_2_/C composite-fiber anode in the MOILE with 5 wt% SN are higher than in the OLE. The improvement in the specific capacity of the composite-membrane anode was attributed to the addition of the high polarity SN to the MOILE and its ability to dissolve the LiTFSI salt, which resulted in enhanced ionic conductivity and improved cycling stability of the electrode in the MOILE. The SnO_2_/C composite-membrane anode in MOILE with 5 wt.% SN shows (Figure 9b) improved cycling stability and capacity retention after the 2nd cycle. 

**Figure 10:**
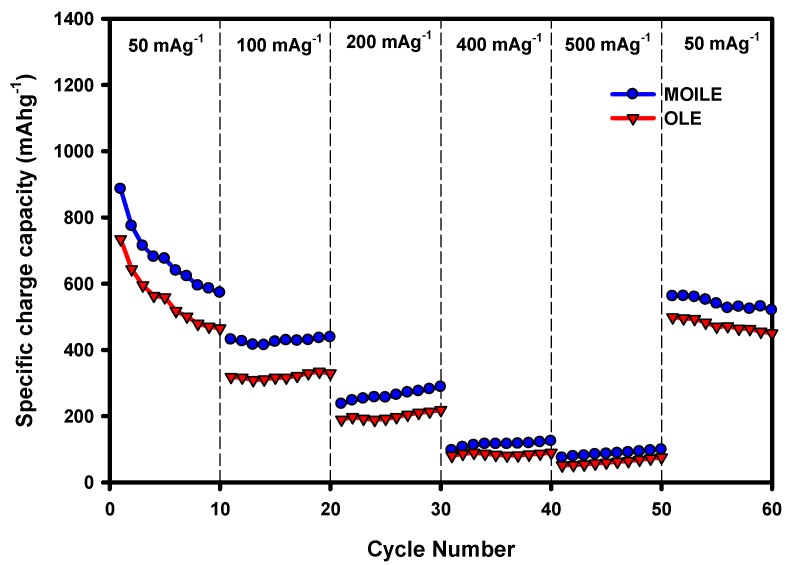
Rate performance (charge capacity vs cycle number at different current densities) of SnO_2_/C composite fibers at 25 °C with two different electrolytes: OLE (1M LiPF_6_ in EC/DMC (1:1 *v*/*v*)), and MOILE (1 M LiTFSI in 60% EMI-TFSI/ 40% EC/DMC (1:1 *v*/*v*) with 5 wt% SN).

The rate performance of the SnO_2_/C composite fibers was further evaluated by conducting current rate (or rate capability) experiments on the lithium anode half-cells at different current densities between 50 and 500 mA g^−1^. The SnO_2_/C composite fibers were cycled ten times at current densities of 50, 100, 200, 400, 500, and then again at the initial value of 50 mA g^−1^ (Figure 10). The results exemplify the SnO_2_/C composite anode’s ability to perform at higher current densities, as well as demonstrating the capacity recovery after being cycled from high to low current density. Figure 10 shows the rate performance (charge capacity vs cycle number at different current densities) of the SnO_2_/C composite-fiber anode in the OLE and in the MOILE with 5 wt.% SN. As expected, the composite-fiber anode delivered a higher specific charge capacity at lower current density, and vice versa. At 50 mAh g^–1^_,_ the specific capacity decreased after 10 cycles to 418 mAh g^–1^ for the Li-ion cell cycled with the 1 M LiPF_6_ in EC/DMC (1:1 *v*/*v*) electrolyte, but only to 579 mAh g^–1^ for the 1 M LiTFSI in 60% EMI-TFSI 40% EC/DMC (1:1 *v*/*v*) with 5 wt% SN electrolytes. This can be attributed to the stresses and strains caused by the high-volume change of the SnO_2_/C composite fibers after repeated charge/discharge cycles. At a current density of 100 mA g^−1^, the charge capacity was stable at ~315 mAh g^−1^ for 1 M LiPF_6_ in EC/DMC (1:1 *v*/*v*) and at ~441 mAh g^−1^ for 1 M LiTFSI in 60% EMI-TFSI 40% EC/DMC (1:1 *v*/*v*) 5 wt% SN. The SnO_2_/C composite anode in the MOILE with 5 wt% SN had a higher percentage increase in specific capacity with 25% at 100 mAh g^−1^, 23% at 200 mAh g^−1^, 30% at 400 mAh g^−1^, and 1% at 500 mAh g^−1^, compared to 1 M LiPF_6_ in EC/DMC (1:1 *v*/*v*). However, after cycling back to 50 mA g^−1^, the SnO_2_/C composite fibers with 1 M LiPF_6_ in EC/DMC (1:1 *v*/*v*) had less specific charge capacity than with the MOILE with 5 wt% SN. However, the SnO_2_ electrode in both electrolytes (OLE and MOILE with SN) shows relatively low capacity at higher current density. Thus, the improvement in the charge capacity of the SnO_2_/C composite anode with MOILE and SN can be attributed to the high Li-ion conductivity and diffusion caused by the addition of SN to the ionic liquid electrolyte. 

## 4. Conclusions

Two different electrolytes, (1 M LiPF_6_ in EC/DMC (1:1 *v*/*v*) and 1 M LiTFSI in 60% EMI-TFSI 40% EC/DMC (1:1 *v*/*v*) with 5 wt% SN), were synthesized, characterized electrochemically, and compared using lithium anode (or cathode) half-cells with either a SnO_2_/C anode or a LiCoO_2_ cathode. The SnO_2_/C composite-fiber electrode was prepared by forcespinning of a PAN/SnO_2_ precursor solution and subsequent thermal treatment. The electrochemical performance results showed that lithium anode half-cells with a SnO_2_/C composite-fiber electrode in 1 M LiTFSI in 60% EMI-TFSI 40% EC/DMC (1:1 *v*/*v*) and 5 wt% SN perform better than that with commercial organic liquid electrolyte. The use of ionic liquid electrolyte with 5 wt% SN in lithium anode half-cells with a SnO_2_/C electrode demonstrated good cycling stability and capacity retention after 100 charge/discharge cycles. The results showed that 1 M LiTFSI in 60% EMI-TFSI 40% EC/DMC (1:1 *v*/*v*) with 5 wt% SN had a higher ionic conductivity than 1 M LiPF_6_ in EC/DMC (1:1 *v*/*v*) electrolyte. The electrochemical performance of a commercial LiCoO_2_ cathode was evaluated at 60 °C using lithium cathode half-cells with MOILEs, both without and with SN electrolytes. The commercial LiCoO_2_ cathode was evaluated electrochemically at 60 °C, cycled with 1 M LiTFSI in 60% EMI-TFSI 40% EC/DMC (1:1 *v*/*v*) and 5 wt% SN and had an excellent performance. The LiCoO_2_ cathode in 60% EMI-TFSI 40% EC/DMC (1:1 *v*/*v*) and 5 wt% SN showed good electrochemical performance at 60 °C, which was attributed to the high ionic conductivity of the MOILE/SN at elevated temperature.
